# Interferon type I signature associated with skin disease in juvenile dermatomyositis

**DOI:** 10.3389/fmed.2024.1214920

**Published:** 2024-02-14

**Authors:** Rinat Raupov, Evgeny Suspitsin, Elena V. Preobrazhenskaya, Mikhail Kostik

**Affiliations:** ^1^Saint-Petersburg State Pediatric Medical University, Saint Petersburg, Russia; ^2^H.Turner National Medical Research Center for Children’s Orthopedics and Trauma Surgery, Saint Petersburg, Russia; ^3^N.N.Petrov Institute of Oncology, Saint Petersburg, Russia; ^4^Research Laboratory of Autoimmune and Autoinflammatory Diseases, World-Class Research Centre for Personalized Medicine, Almazov National Medical Research Centre, Saint Petersburg, Russia

**Keywords:** inflammatory myopathy, juvenile dermatomyositis, IFN-I signaling pathway, interferon score, interferon type I signature

## Abstract

**Background:**

Interferon type I (IFN-I) signaling system hyperactivation plays an important role in the pathogenesis of juvenile dermatomyositis (JDM).

**Aim of the study:**

To analyze IFN-I score with disease activity in patients with JDM.

**Materials and methods:**

Clinical manifestations laboratory data, and treatment options were analyzed in 15 children with JDM. Disease activity was assessed by CMAS (childhood myositis assessment tool) and CAT (cutaneous assessment tool) scores. IFN I-score was assessed by RT-PCR quantitation of 5 IFN I-regulated transcripts (IFI44L, IFI44, IFIT3, LY6E, MXA1).

**Results:**

All patients had skin and muscle involvement, some had a fever (*n* = 8), swallowing disorders (*n* = 4), arthritis (*n* = 5), calcinosis (*n* = 3), lipodystrophy (*n* = 2), and interstitial lung disease (*n* = 5). Twelve patients had elevated IFN I-score and it was correlated with skin disease activity. Ten patients had clinically active disease and the level of IFN I-score and its components were higher than in patients with inactive disease (8.8 vs. 4.2, *p* = 0.011). IFN I-score was evaluated in nine patients during follow-up. The simultaneous reduction of IFN I-score and its components, CMAS and CAT scores was observed.

**Conclusion:**

Skin involvement in refractory JDM is a challenging problem requiring the use of additional medications. Serum IFN I-score might be suggested as the promising biomarker of skin disease activity in JDM patients. Further investigations on patients with JDM and recurrent disease activity are needed, especially concerning biomarkers that determine the response to JAK inhibitors and treatment options for patients who don’t respond to them.

## Introduction

Juvenile dermatomyositis (JDM) belongs to a group of idiopathic inflammatory myopathies (IIM), affecting children until 16 years and characterized by muscle involvement with skin vasculopathy (1–3). The etiology of JDM is still unclear. Hyperactivation of the interferon-I signaling system is one of the key moments of pathogenesis, as well as the production of auto-antibodies, however, 30–40% of patients do not have auto-antibodies, which indicates other mechanisms of disease development (4, 5).

There are several sets of JDM classification criteria proposed by Bohan **И** Peter (1976), Tanimoto (1995), and ACR/EULAR (2017), but Tanimoto criteria predominantly uses for JDM diagnostics (6–8). Children have a higher prevalence of dermatomyositis, calcinosis, and lipodystrophy while adults are characterized by the prevalence of different subtypes of IIM, higher rate of lung and myocardial involvement, and antisynthetase autoantibodies (9).

Several special scores—CMAS (childhood myositis assessment tool) and MMT-8 (manual muscle testing) are used for the assessment of muscle involvement (10, 11) as well as CAT (cutaneous assessment tool), and CDASI (cutaneous disease area and severity index) are used for skin disease (12, 13).

There are no validated biomarkers for the assessment of JDM activity. Nowadays neopterin, CXCL11, and galectin-9 are considered as the most perspective biomarkers for JDM (14).

The ultrasound is a promising tool for assessment of skin disease in patients with connective tissue diseases (15–17).

Interferon (IFN) signature is a surrogate biomarker of IFN signaling cascade hyperactivation. It might be assessed in different tissues, e.g., blood, skin, and muscles. IFN type I and chemokine profile depend on the subtype of inflammatory myopathies (18, 19).

Patients with monocyclic JDM are good responders for traditional treatment with corticosteroids and methotrexate. Otherwise, the management of patients with polycyclic course or recurrent skin disease is still challenging for pediatric rheumatologists. Different treatment options have been used in such cases including JAK inhibitors blocking activation of IFN type I signaling system (17). In several trials and studies of agents inhibiting the IFN-I signaling pathway in IIM, the IFN signature was used as the biomarker of efficacy (20–22).

*This study* aimed to analyze the association between serum IFN-I score and signs of disease activity in children with JDM.

## Methods

### Patients

In the cohort study, 15 children (10 girls and 5 boys) with JDM from different parts of Russia have been included (nine patients are from St Petersburg, five patients are from the central area of Russia, and one patient is from the southern part of Russia).

### JDM diagnosis and disease activity assessment

The diagnosis was made by Tanimoto criteria (7). Clinical and laboratory parameters and treatment options were evaluated. Disease activity was assessed by CMAS (childhood myositis assessment tool) and CAT (Cutaneous Assessment Tool) scores (11, 12). Muscle and skin disease activity parameters (CMAS, CAT) have not been evaluated in the disease onset.

### IFN signature assessment

Whole blood was collected in Tempus™ Blood RNA tubes. Total RNA was extracted from blood leukocytes using Tempus RNA Isolation Kit according to the manufacturer’s instruction. cDNA was subjected to reverse transcription (RT). RT reaction (final volume of 20 uL) contained 5X reverse transcriptase reaction buffer, 200 U of RevertAid Reverse Transcriptase (Thermo Fisher Scientific Baltics UAB, Vilnius, Lithuania), 20 U of RiboCare RNase Inhibitor (Evrogen, Moscow, Russia), dNTP mix (20 nM each), random hexamers (0.25 μmol). The mixture of RNA, dNTPs, and primers was consecutively incubated for 5 min at 70, 65, and 60°C to achieve primer annealing, and then cooled at 0°C for 2 min; after the adding of the enzymes the reaction mix was incubated at 20°C for 5 min, 38°C for 30 min and 95°C for 5 min. A total of 40 uL of sterile water was added; 1 uL of cDNA solution was used for qPCR. PCR reaction contained 1X GeneAmp PCR Buffer I (Applied Biosystems, USA), 250 mkM of each dNTP, 200 nM of each primer and probe, 2.5 mM MgCl2 and 1U of TaqM-polymerase (AlkorBio, Russia) in a final volume of 20 uL. The following forward (f) and reverse (r) primer and probe (p) sequences were used for quantitative real-time PCR:

ifi44l_f1 ACTGTGCATGGATGACATTCC, ifi44l-r CAGGTG TAATTGGTTTACGGGAA, ifi44l_p FAM-TAAACTGATATC TGTCTGGCATACAACCTT-BHQ1, ifi44_f GAAAGAAAGAT AAAAGGGGTCATTG, ifi44_r CCATATGGTTCATAAGTTCTC AAGG, ifi44_p FAM-TCAGGAAGAGCTTACTGTCTGCCTTGA-BHQ1, ifit3_f GAACAAATCAGCCTGGTCAC, ifit3_r GAA GGATTTTCTCCAGGGAATTC, ifit3_p FAM-AACAGCAGAGA CACAGAGGGCAGTCAT-BHQ1, ly6e-f CTGCTGGTACCTG CGTCC, ly6e-r CATTCTGGAGAGGATGGCCG, ly6e_p FAM-TCACAAACCAAAGCAGCCTGTCCT-BHQ1, mx1_f CTGAATGGAGATGCTACTGTGG, mx1_r CACCTTCTCC TCATACTGGCTG, mx1_p FAM-TTGTTCTCAGCCACCGAG CCT-BHQ1, sdha_f CCACTCGCTATTGCACACC, sdha_r ATCCAAGGCAAAATACTCCAC, sdha_p R6G-CTGGTATC ATATCGCAGAGACC-BHQ2.

PCR reaction was performed using Bio-Rad CFX96 machine; conditions included enzyme activation step (10 min at 95°Ñ) followed by 50 cycles of amplification (15 s at 95°Ñ, 20 s at 58°Ñ, 30 s at 72°Ñ). Relative expression was analyzed using Bio-Rad Gene Expression software. The samples were normalized against the expression of the household SDHA gene. Fold change values were determined using the 2^–Δ^ ^Δ^ *^CT^* method.

Interferon type signature was measured by quantitation of 5 IFN I-regulated transcripts (*IFI44*, *IFI44L*, *IFIT3*, *LY6E*, *MX1*). To determine the normal range of IFN-I score values we previously analyzed RNA samples from 30 clinically healthy individuals who do not have a history of a recent infectious disease. The IFN-I score in this group ranged from 0.5 to 1.9 (median 1.2); the value of ≥2 was considered a diagnostic threshold indicative of IFN I-pathway hyperactivation. Samples from patients with a genetically confirmed diagnosis of interferonopathy (DADA2 and SAVI syndromes) and known IFN-I scores measured in another lab were used as positive controls.

In nine patients IFN-signature was evaluated repeatedly. The analysis was done in the following subgroups: (i) active (*n* = 10) and non-active (*n* = 5) JDM patients; and (ii) JDM patients with elevated (*n* = 12) and normal (*n* = 3) IFN I-score.

The patients were divided into 2 groups according to IFN-I score (high and normal) and disease activity (active and non-active).

### Statistics

The sample size was not calculated. The software Statistica (release 10.0, StatSoft Corporation, Tulsa, OK, USA), Biostat, and MedCalc were used for the data analyses.

All continuous variables were checked by the Kolmogorov-Smirnov test: no normal distribution was identified. The descriptive statistics were reported in medians and interquartile ranges (IQRs) for continuous variables and absolute frequencies and percentages for categorical variables.

We used the Mann-Whitney *U*-test to compare two independent quantitative variables and the chi-square test for the comparison of two categorical variables, or the Fisher’s exact test in case of expected frequencies <5.

A comparison of two dependent quantitative variables was carried out using Wilcoxon’s matched paired test and the Mac-Nemar test was applied for dependent categorical variables.

Spearmen correlation analysis between categorical and quantitative variables was performed.

A *p*-value of less than 0.05 was considered statistically significant.

## Results

### Patients’ demography

The median age of inclusion in the study was 8.8 (5.7; 10.8) years while the median age of the disease onset was 6.2 (3.6; 7.6) years. All patients had skin (Gottron’s papules, face erythema) and muscle involvement at the onset of the disease (Table 1). Active disease status was in 10 patients and 5 patients were inactive at the time of inclusion in the study. Insolation triggered JDM in 5/15 patients and two of these five patients had sun exposure during seaside vacations with a duration of about 1–2 weeks and acute infection in 3/15 (20%). In the remaining patients, the trigger was not identified.

**TABLE 1:** Characteristics of patients with JDM at the study inclusion.

Parameter	Results
The median age of inclusion, years, Me (25%; 75%)	8.8 (5.7; 10.8)
The median age of JDM onset, years, Me (25%; 75%)	6.2 (3.6; 7.6)
**Trigger, *n* (%) patients**	
Insolation	5 (33)
Acute infections	3 (20)
Unknown	7 (47)
**Clinical manifestations in the onset and disease** **development, *n* (%) patients**	
Muscle involvement	15 (100)
Gotton’s papules	15 (100)
Face erythema	15 (100)
Heliotrope rash	14 (93)
Body erythema	9 (60)
Livedo reticularis	9 (60)
Skin ulceration	3 (20)
Fever	8 (53)
Cheilitis	6 (40)
Stomatitis	2 (13)
Difficulty in swallowing	4 (27)
Arthritis	5 (33)
Calcinosis	3 (20)
Lipodystrophy	2 (13)
Hepatomegaly	7 (47)
Splenomegaly	3 (20)
Interstitial lung disease	5 (33)
**Myositis confirmation, *n* (%) patients**	
US or MRI	9 (60)
Electroneuromyography	4 (27)
Muscle biopsy	1 (7)
**Laboratory in the disease onset, *n* (%) patients**	
Leucopenia	2 (13)
Elevated CRP/ESR	9 (60)
High ALT	9 (60)
High AST	13 (87)
High LDH	15 (100)
High CK	10 (67)
ANA positivity	10 (67)
Autoantibodies:	2 (13)
SRP	4 (27)
SAA	2 (13)
PM-Scl	1 (7)
Jo-1	2 (13)
CENP-B	1 (7)
Ku	
**Treatment, n (%) patients**	
- Pulse methylprednisolone	14 (93)
- Intravenous immunoglobulin	14 (93)
- Prednisone	15 (100)
- Methotrexate	15 (100)
- Mycophenolate mofetil	3 (20)
- Cyclosporine A	2 (13)
- Cyclophosphamide	1 (7)
- Azathioprine	1 (7)
- Tofacitinib	3 (20)
- Baricitinib	1 (7)

ANA, antinuclear antibodies; ALT, alanine aminotransferase; AST, aspartate aminotransferase; CRP, C-reactive protein; CK, creatine kinase; ESR, erythrocyte sedimentation rate; LDH, lactate dehydrogenase; Me, median; MRI, magnetic resonance imaging; US, ultrasound.

At the disease onset, all patients (100%) had muscle involvement, Gotton’s papules, and face erythema. Other skin involvement included heliotrope rash (93%), body erythema (60%), livedo reticularis (60%), skin ulceration (20%), calcinosis (20%), and lipodystrophy (13%). Interstitial lung disease had five (33%) patients. All patients initially received the standard of care treatment with corticosteroids (intravenous, followed oral) and methotrexate. Intravenous immunoglobuline received 93% of patients. Three patients (20%) with refractive skin disease, failed standard of care treatment and IVIG received JAK-inhibitors.

### Interferon I score assessment and its association with disease activity

Muscle and skin disease activity parameters (CMAS, CAT) have not been evaluated in the disease onset. The elevated IFN I-score was in 12 (80%) patients. There was no difference in laboratory parameters between patients with normal and elevated IFN I-score. Median CMAS was 35 (23; 42) units in patients with elevated and 46 (40; 48) units with normal IFN I-score (*p* = 0.07).

Active skin disease was only in patients with elevated IFN I-score: CAT score was 3 (2; 6) points in patients with elevated IFN I-score compared with patients with normal IFN I-score–0 (0; 0) points (*p* = 0.004). The levels of CMAS and CAT depending on the IFN-I score level are in Table 2. Ten patients had active disease. Comparison between patients with active and non-active diseases revealed differences in IFN I-score and its components, in laboratory parameters and activity scores (Table 3).

**TABLE 2:** Dynamics of the disease activity scores in patients depending on IFN-I scores during the study.

Assessed parameters (baseline)	Whole group (*n* = 15)	IFN-I score, high (*n* = 12)	IFN-I score, normal (*n* = 3)	*p*-value
CMAS, units, Me (25%; 75%)	38 (28; 46)	35 (23; 42)	46 (40; 48)	0.07
CAT-activity, units, Me (25%; 75%)	3 (1; 5)	3 (2; 6)	0 (0;0)	0.004
CAT-damage, units, Me (25%; 75%)	0 (0; 1)	0 (0; 1)	1 (1; 1)	0.101
**JDM activity changes (2nd assessment,** **changes since baseline)**	**Decreasing in IFN** **score**	***p*-value**	**Δ IFN score**	***p*-value**
Δ CMAS, points	*r* = −0.116	0.767	*r* = 0.339	0.372
The improvement of CMAS, yes	*r* = 0.189	0.626	*r* = 0.300	0.433
Δ aCAT, points	*r* = 0.105	0.788	*r* = 0.688	0.041
The improvement of aCAT, yes	*r* = 0.286	0.456	*r* = 0.821	0.007
The improvement of dCAT, yes	*r* = 0.357	0.345	*r* = 0.444	0.231

CAT, cutaneous assessment tool; aCAT, CAT activity score; CAT, CAT damage score; CMAS, childhood myositis assessment tool; Δ, delta, difference between two measurements; IFN, **i**nterferon.

**TABLE 3:** IFN I-score in patients depending on the JDM disease activity.

Parameters	Active disease (*n* = 10)	Non-active disease (*n* = 5)	*p*-value
IFN I-score, total, Me (25%; 75%)	13.6 (8.9; 24.7)	1.4 (1.4; 2.0)	0.006
IFI44, Me (25%; 75%)	30.5 (18.3; 42.1)	2.6 (1.4; 2.9)	0.006
IFI44L, Me (25%; 75%)	48.6 (27.2; 87.5)	2.3 (2.2; 3.1)	0.009
IFIT3, Me (25%; 75%)	11.7 (5.1; 15.9)	1.0 (0.9; 1.5)	0.006
LY6E, Me (25%; 75%)	10.2 (4.7; 23.6)	1.3 (1.0; 1.3)	0.012
MX1, Me (25%; 75%)	12.2 (6.1; 20.3)	1.5 (1.4; 2.0)	0.006
ESR, mm/h, Me (25%; 75%)	12 (6; 15)	2 (2; 2)	0.004
ALT, UE/ml, Me (25%; 75%)	35 (23; 124)	15 (12; 16)	0.037
LDH, U/l, Me (25%; 75%)	364 (272; 461)	(163; 233)	0.024
CMAS, Me (25%; 75%)	34 (18; 38)	46 (40; 48)	0.019
CAT activity, Me (25%; 75%)	4 (3; 6)	0 (0; 1)	0.001

CAT, cutaneous assessment tool; aCAT, CAT activity score; CMAS, childhood myositis assessment tool; ESR, erythrocyte sedimentation rate; LDH, lactate dehydrogenase.

### Follow-up interferon type I assessment

Interferon type I-score has been measured in nine patients during follow-up. During the study IFN I-score decreased in 7/9 (78%) patients, CAT score in 7/9 (78%) patients and CMAS increased in 8/9 (89%) patients. In all patients with skin disease activity CAT score and IFN I-score reduction were observed (*r* = 0.687; *p* = 0.041) (Supplementary Figure 1 and Table 2). Positive correlations between IFN I-score, its components, and disease activity, CAT activity, cheilitis, and arthritis were observed (Table 4).

**TABLE 4:** Correlation between IFN-I score (r) and its components with activity and symptoms of JDM.

Presented parameter	IFN-score at baseline	IFN-score follow-up	IFI44	IFI44L	IFIT3	LY6E	MX1
Disease activity	0.577*	0.707*	0.637*	0.643*	0.54*	0.541*	0.570*
aCAT	0.498	0.691*	0.548*	0.457*	0.525*	0.449	0.551*
dCAT	−0.194	−0.522*	−0.352	−0.199	−0.312	−0.114	−0.287
Cheilitis	0.458	0.408	0.352	0.488*	0.440	0.524*	0.361
Arthritis	0.999*	0.500	0.999*	0.999*	0.999*	0.999*	0.999*

aCAT, cutaneous assessment tool activity score; dCAT, cutaneous assessment tool damage score; IFN, interferon.

*p < 0.05.

### Treatment with JAK-inhibitors

Three patients with refractory skin disease were treated with tofacitinib. All patients had an increased IFN-I score. One patient achieved complete remission (no skin and muscle disease activity) with normalization of IFN-I score and the remaining two patients had a partial response to tofacitinib. They had an initial improvement of skin disease, followed by a flare when prednisone tapered less than 0.2 mg/kg. In both of these patients have decreased IFN-I score, but its normalization could not be achieved.

## Discussion

We performed indirect measurements of type I interferon activity using relative expression levels of five IFN I-stimulated genes previously used in JDM patients (23, 24). The exact functions of molecules encoded by the corresponding genes and their role in JDM pathogenesis are unclear; the expression of these IFN I–I-stimulated transcripts is used as a surrogate marker of IFN I signature.

Our study supports previous results that the IFN I-score is associated with skin activity and could be used as a skin disease biomarker. In our group of patients, a decrease in IFN I-score corresponded with a decrease in disease activity. A correlation between IFN I-score and arthritis was found.

### Interferon type I hyper-activation in JDM pathogenesis

The role of interferons in the development of dermatomyositis has been studied for the last 10–15 years. The majority of these studies demonstrated hyperactivation of the IFN I signaling pathway in blood, muscle, and skin tissue in patients with dermatomyositis (4).

Baechler et colleagues analyzed IFN-inducible gene and IFN chemokine scores in adult and pediatric patients with dermatomyositis. Both groups of patients had higher scores than healthy controls. The correlation of IFN gene score with disease activity has been demonstrated only in adult patients, while IFN chemokine score correlated with muscle disease activity and global VAS in adults and children (25). The positive correlation of IFN type I signature in blood with disease activity in the majority of patients with dermatomyositis and polymyositis was observed, except in the patients with inclusion body myositis (26).

The assessment of IFN signature might help distinguish between subtypes of IIM in adults. IFN I-signature in muscles was associated with dermatomyositis, whereas IFN- γ signature inclusion body myositis and antisynthetase myositis (18).

The changes in interferon chemokine score (IP-10, MCP-1) corresponded with changes in extra muscular disease activity score during two subsequent visits in 20 children with JDM (27).

Interferon type I score based on measuring 28 transcript expression profiles was compared between patients with JDM and monogenic type I interferonopathies (CANDLE, SAVI). Patients with JDM had higher IFN I scores than healthy controls, but lower than patients with interferonopathies. High IFITI expression led to the elevation of IFN I-score in JDM than in SAVI and CANDLE. IFN I-score moderately correlated with JDM disease activity scores (physician global activity (PGA), manual muscle testing (MMT), extra-muscular global and skeletal activity, and Disease Activity Score–DAS) (28).

### Interferon type I blockade with JAK-inhibitors

Several studies demonstrated the efficacy of JAK inhibitors in patients with pediatric and adult dermatomyositis. IFN I-score could be considered as a biomarker of treatment efficacy. Four adult patients with refractory dermatomyositis (remained active disease after initiating of two different immunosuppressive drugs with corticosteroids with or without immunoglobulin) and elevated IFN I-score received ruxolitinib up to 40 mg per day for 3 months. Clinical remission (no skin and muscle disease activity) and IFN I-score reduction were reported in all patients (29).

Facial skin rash and CDASI score improved in all four patients and muscle strength improved in patients with clear muscle weakness and creatine kinase levels also decreased significantly in one of them. All patients reported an improvement in their quality of life score. Ruxolitinib decreased IFN levels and interferon-stimulated genes score in PBMCs (29).

Tofacitinib treatment allowed for achieving clinical improvement (increased CMAS from 18 to 40 points) and reduction of IFN I-score in JDM patients with persistent disease activity and high IFN I-score (30). During treatment with tofacitinib 11 mg/day of adults and children with refractory dermatomyositis, 50% of the patients experienced moderate improvement and 50% had minimal improvement and the mean change in the CDASI activity score over 12 weeks was statistically significant (since 28 ± 15.4 at baseline vs. 9.5 ± 8.5 at 12 weeks) (*p* = 0.0005) with the decreasing of serum chemokine levels of CXCL9/CXCL10 from baseline was demonstrated (31).

Our previous experience demonstrated the high efficacy of tofacitinib in one of two patients with refractory JDM. Tofacitinib controlled skin disease and allowed discontinued corticosteroids (32).

### Study limitations

The study limitations are related to the small number of patients, heterogeneity of the studied population according to disease activity and disease duration, and absence of testing the most frequent autoantibodies for JDM (anti-TIF1 and anti-NXP2). The absence of the clinical and interferon type I score assessment (CMAS, CAT) in the disease onset influences study results. Previous treatment (pre-assessment of interferon type I score) may misrepresent study results.

## Conclusion

Skin involvement is refractory JDM is a challenging problem requiring using additional medications. Hyperactivation of the IFN I-signaling system in JDM patients was observed. Serum IFN I-score might be suggested as the promising biomarker of skin disease activity in JDM patients. Further investigations on patients with JDM and recurrent disease activity are needed, especially concerning biomarkers that determine the response to JAK inhibitors and treatment options for patients who don’t respond to them.

## Data availability statement

The datasets generated during and/or analyzed during the current study are available from the corresponding author upon reasonable request.

## Ethics statement

The studies involving humans were approved by the Ethics Committee of Saint-Petersburg State Pediatric Medical University approved the study (protocol # 1/3 îò 11.01.2021). The studies were conducted in accordance with the local legislation and institutional requirements. Written informed consent for participation in this study was provided by the participants’ legal guardians/next of kin.

## Author contributions

All authors were involved in drafting the article or revising it critically for important intellectual content, and approved the final version to be published.

## Supplementary material

Supplementary Figure 1 The dynamics of IFN I-score and JDM activity scores measurement during the study.
