# Supplementary figures and images for: Interferon type I signature associated with skin disease in juvenile dermatomyositis
Source: Front Med (Lausanne). 2024 Feb 14;11:1214920. doi: 10.3389/fmed.2024.1214920 (PMC10899462; doi:10.3389/fmed.2024.1214920)

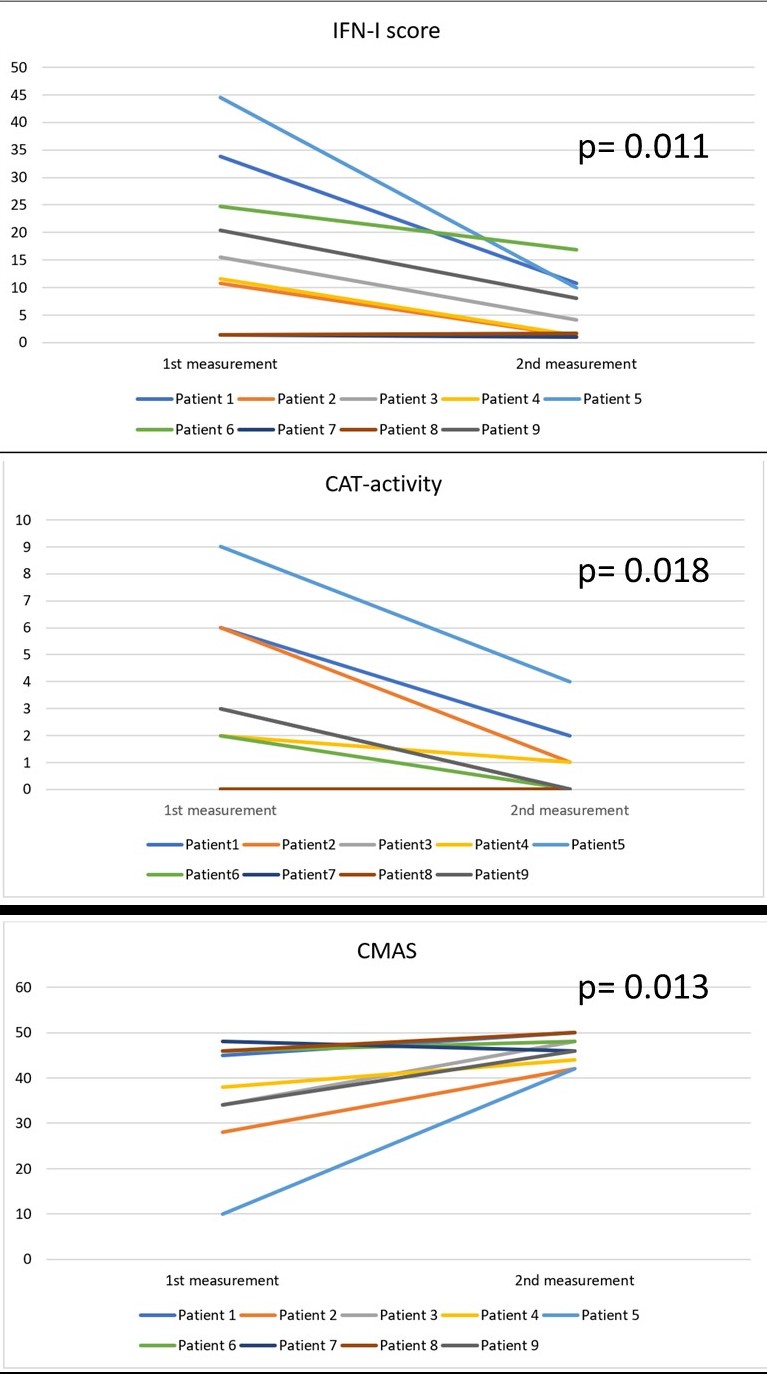

Supplement: Supplementary Figure 1 — The dynamics of IFN I-score and JDM activity scores measurement during the study. [file Image_1.JPEG]
